# Level and determinants of food insecurity in East and West Gojjam zones of Amhara Region, Ethiopia: a community based comparative cross-sectional study

**DOI:** 10.1186/s12889-016-3186-7

**Published:** 2016-06-11

**Authors:** Achenef Motbainor, Alemayehu Worku, Abera Kumie

**Affiliations:** School of Public Health College of Health Science, Addis Ababa University, P. O. Box: 23676, Code: 1000 Addis Ababa, Ethiopia

**Keywords:** Food security, Food insecurity, Gojjam, Amhara, Ethiopia

## Abstract

**Background:**

Food insecurity remains highly prevalent in developing countries and over the past two decades it has increasingly been recognized as a serious public health problem, including in Ethiopia. An emerging body of literature links food insecurity to a range of negative health outcomes and causes of a decline in productivity. The objectives of the present study were to determine the level of food insecurity in East Gojjam zone where the productive safety net program is available, and in West Gojjam zone where there is no program, and to identify the determinants of food insecurity in both East and West Gojjam zones of Amhara Region, Ethiopia.

**Methods:**

Community based comparative cross-sectional study design was used from 24 May 2013- 20 July 2013. Multistage sampling technique was implemented. A total of 4110 randomly selected households in two distinct populations were approached to be included in the study. Availability and absence of the productive safety net program between the two study areas was used to categorize them as comparative groups; otherwise the two communities are comparable in many socio-cultural characteristics. The household food security access scale questionnaire, developed by the Food and Nutrition Technical Assistant Project, was used to measure food security level. Socio-demographic and other household level information were collected by using a structured questionnaire. The binary logistic regression model was used to assess factors associated with food insecurity.

**Results:**

From the total 4110 households, 3964 (96.45 %) gave complete responses. The total prevalence of food insecurity was 55.3 % (95 % CI: 53.8, 56.8). To compare food insecurity levels between the two zones, nearly sixty percent, 59.2 % (95 % CI: 57 %, 61.4 %) of the East Gojjam and 51.3 % (95 % CI: 49.1 %, 53.5) of West Gojjam households were food insecure.

Family size (2–4) (AOR = 0.641, 95 % CI: 0.513, 0.801), non-merchant women (AOR = 1.638, 95 % CI: 1.015, 2.643), household monthly income quartiles, 1^st^ (AOR = 2.756, 95 % CI: 1.902, 3.993), and 2^nd^ (AOR =1.897, 95 % CI: 1.299, 2.775) were the significant socio-demographic determinants in east Gojjam zone. Illiterate mothers (AOR = 1.388, 95 % CI: 1.011, 1.905), household monthly income quartiles, 1^st^ (AOR = 3.110232, 95 % CI: 2.366, 4.415), 2^nd^ (AOR =2.618, 95 % CI: 1.892, 3.622) and 3^rd^ (AOR = 2.177, 95 % CI: 1.6911, 2.803) were the significant socio-demographic predictors in west Gojjam zone.

Rural residential area (AOR = 3.201, 95 % CI: 1.832, 5.594) and (AOR = 2.425, 95 % CI: 1.79, 3.272), highland agro-ecology (AOR = 2.193, 95 % CI: 1.348, 3.569 and AOR = 3.669, 95 % CI: 2.442, 5.513) and lack of livestock (AOR = 1.553, 95 % CI: 1.160, 2.078 and AOR = 1.568 95 % CI: 1.183, 2.080) were significant environmental predictors in east and west Gojjam zones respectively.

**Conclusion:**

Food insecurity is highly prevalent in both study areas; however, there are different predictor factors. Intervention strategies should give emphasis to women’s education, diversified income generating opportunities, and for each agro-ecological zone, mixed agriculture strategy.

## Background

Food insecurity is a state or a condition in which people experience limited or uncertain physical and economic access to safe, sufficient and nutritious food to meet their dietary needs or food preferences for a productive, healthy and active life [1–4]. Food security can be considered at national, household and individual levels. At national level, it is related to physical existence of food stocks for consumption be it from own production or from markets [5]. It is related to the availability dimension of food security and is a function of the combinations of domestic food stocks, commercial food imports, food aid and domestic food production including determinants of each of these factors [5]. On the other hand household food security is related to the ability to obtain sufficient food with sufficient quality to meet nutritional requirements of all household members. Household level food security mainly relies on economic freedom and purchasing power of household members which again related to income distribution in the household [5, 6].

Although non-availability of food, lack of access, improper utilization and instability over a certain period time are the four main pillars that lead to a situation of food insecurity, it exists in various ways in different parts of the world [7]. Limited resource and increased food price problems affecting many households of the world including Ethiopia, are the common factors that affect food insecurity [8, 9]. In addition to the basic causes of food insecurity, literature showed that the educational status of the household, multiple income sources, number of children, sole parenthood, marital status and employment status of the households are perceived to be determinants of food insecurity both in developed and developing countries [9–11].

Moreover, traditional farming practice, unstable weather conditions, recurrent drought, pests and disease, population pressure or growth, weak institutional capacity, inadequate infrastructure and social services are the other major reasons that determine food security in Ethiopia [12].

Food insecurity remains highly prevalent in developing countries and over the past two decades, it has increasingly been recognised as a serious public health problem in the developed world [11]. According to the latest estimates of the State of Food Insecurity in the World (2015 report), Sub-Saharan Africa made some progress towards halving the proportion of its population suffering from hunger. The overall prevalence of hunger in the region declined by 30 % between 1990-92 and 2015 [13]. This progress has been made with significant differences between the four sub-regions of Sub-Saharan Africa, with 60 % reduction in Western Africa and with no progress in the Middle Africa. Since 1990-92, other sub-regions experienced an increase in the absolute number of undernourished people, approximately 20 % and 2 % respectively in Eastern and Southern Africa [5, 13]. Thus, the FAO estimated that about 805 million people are chronically undernourished in 2012-14, down more than 100 million over the last decade and 209 million lower than in 1990-92. In the same period, the prevalence of undernourishment has fallen from 18.7–11.3 % globally and from 23.4–13,5 % for developing countries [5, 14].

Drought and famine have become an everyday reality in Ethiopia. The country has faced three major famines and other similar situations in the past three decades [5]. The recurrence of famine in the 1970s, 1980s and 1990s has significantly affected the country’s food production [5, 13]. However, during the past 20 years, the deficit in calorie intake in Ethiopia has been significantly reduced from 623 to 314 Kcal/cap/day accompanied by a 33 % decline in poverty rate from 1999 to 2010 [13, 15]. Although these results are recorded as good progress in poverty reduction in Ethiopia, food insecurity is still a threat to the households of the country [15].

Studies conducted at small scale level in different parts of Ethiopia showed that almost 50 % of the study populations were food insecure. According to the cross-sectional study done in Farta district (Ethiopia) in 2012, from the total study participants about 70.70 % were food insecure [16]. Similarly, a study done in Addis Ababa city showed 58.16 % of the total households were below the food security cutoff point that classified food secure and insecure households and expressed this in terms of caloric requirements [12, 17]. Another study result from Sidama, Southern Ethiopia, showed 54.10 % of the households were food insecure [18]. A longitudinal study done in Ethiopia on adolescents’ food security status has shown that different or fluctuating levels of food insecurity were registered in different rounds of the study period. Overall, 20.50 % of adolescents were food insecure in the first round survey, while the proportion of adolescents with food insecurity increased to 48.40 % one year later. During the one year follow up period, more than half (54.80 %) of the youth encountered transient food insecurity [8].

Food insecurity has been linked in the literature to a variety of health outcomes such as: undernutrition, iron deficiency anemia, multiple chronic conditions, obesity and poor self-rated physical and mental health [9, 19]. It is also perceived that food insecurity results in social deprivation within household members which further causes different health disorders [20, 21].

In general, Ethiopia has made substantial progress in poverty reduction in the last two decades. However, food insecurity is a threat to households as result of events such as population growth, food prices and recurrent drought. In 2004, the government of Ethiopia initiated a food security strategy built around: increasing the availability of food through domestic production, ensuring access to food for food deficient households, and strengthening institutional emergency response capability [22]. This is the Productive Safety Net Program, which has been conducted in Ethiopia in different phases- phase I (2005-2006), phase II (2007-2009) and phase III (2010-2014)- since its inception in 2005 and is expected to result in improvement of household living and reduced food insecurity levels [13, 23]. The scientific community needs to establish reliable monitoring and evaluating systems to determine whether there is improvement in household food security level as an indicator of poverty. Therefore, the present study determined the levels and associated predictors of food insecurity in the east and west Gojjam zones of Amhara Region, Ethiopia. Policy makers and development agencies working on food security intervention can use the evidence generated from the current study as a baseline for their monitoring and evaluation activities.

## Methods

### Study area and period

The study was conducted in Amhara Regional State which covers some 157,647 km^2^ across north western and eastern Ethiopia and has a total population of 20,018,999 (10,011,795 males and 10,007,204 females) from 24 May 2013- 20 July 2013 [24, 25]. The region is divided in to a number of highland areas separated by deep river valleys, and the eastern and western escarpments and their associated lowlands [25]. Specifically the study was conducted in the east and west Gojjam zones of the region. East Gojjam zone, which is located in the northwest 300 km distance from Addis Ababa, has 2,451,959 total population (1,199,952 males and 1,252,006 females). West Gojjam zone, which is located in the same direction at 385 km from Addis Ababa, has a total population of 2,474,254 (1,220,477 males and 1,253,777 females) [24]. The mean annual temperature of the region ranges from 22-27^O^C in the lowlands and between 10 and 22^O^C in the highlands up to 3,000 meter above sea level [25]. The long term mean annual rainfall of the region is 1165.2 mm [26]. However areas in the specific study sites received 1100 to 1360 mm of mean annual rainfall per year [26]. Within the region four major cereal systems have been recognized: sorghum-maize system in the lowland agro-ecological zone, wheat-teff system in the single rain season area of the mid-land agro-ecological zone, wheat-teff system in the double rain seasons of the mid-land agro-ecological zone and barley system in the high land agro-ecological zone [25].

### Study design, sample size determination and sampling techniques

Community based comparative cross-sectional study design was used to determine the level of food insecurity and its determinants. Households in the study area were used as a sampling unit and all the necessary data were drawn from the mother in the household. The two groups were classified based on the availability of the productive safety net program; Group 1 with the productive safety net program and Group 2 without the productive safety net program. The current study used a sample size determined for another larger study that aimed to see the association between food insecurity and malnutrition. Although the study concerns for stunting, wasting and underweight, the prevalence of stunting has been taken to determine the sample size as it is considered to be the best feature of nutritional status of the community and also since it is not affected by acute events. The 2011 EDHS national prevalence (44 %) has been taken as the malnutrition prevalence for food surplus area and 50 %, which is the worst, for food insecure area as there is no specific study for this area. The study is designed to show the difference at the significance level of 1 % and power of 90 %.

$$ \mathbf{n}=\frac{{\left[{\boldsymbol{z}}_{\boldsymbol{\alpha}}\sqrt{2\boldsymbol{p}\left(1-\boldsymbol{p}\right)} + {\boldsymbol{z}}_{1-\boldsymbol{\beta}}\sqrt{{\boldsymbol{p}}_1\left(1-{\boldsymbol{p}}_1\right)+{\boldsymbol{p}}_2\left(1-{\boldsymbol{p}}_2\right)}\ \right]}^2}{{\left({\boldsymbol{p}}_1-{\boldsymbol{p}}_2\right)}^2} $$

Where*,* P_1_ is the prevalence of stunting in Ethiopian children underfive;

$$ \begin{array}{l}{\mathrm{P}}_2\mathrm{is}\ \mathrm{t}\mathrm{he}\ \mathrm{a}\mathrm{ssumed}\ 50\ \%\ \mathrm{prevalence}\ \mathrm{o}\mathrm{f}\ \mathrm{stunting}\ \mathrm{in}\ \mathrm{underfive}\ \mathrm{children}\ \mathrm{f}\mathrm{o}\mathrm{r}\ \mathrm{f}\mathrm{o}\mathrm{o}\mathrm{d}\ \mathrm{in}\mathrm{secure}\ \mathrm{a}\mathrm{r}\mathrm{ea};\hfill \\ {}\mathrm{P} = {\mathrm{P}}_1 + {\mathrm{P}}_2/2 = \left(0.44 + 0.5\right)/2 = 0.47;\hfill \\ {}{\mathrm{Z}}_{\upalpha /2}\mathrm{a}\mathrm{t}\ 1\ \%\ \mathrm{significance}\ \left(\mathrm{Z}0.01\right) = 2.58;\ \mathrm{a}\mathrm{nd}\hfill \\ {}1\hbox{-} \upbeta \mathrm{a}\mathrm{t}\ 90\ \%\ \mathrm{power}\ \left(\mathrm{Z}1\hbox{-} 0.9\right) = 1.28.\hfill \end{array} $$

Therefore, the sample size will be;

$$ \boldsymbol{n}=\frac{{\left[2.58\sqrt{2\times 0.47\left(1-0.47\right)} + 1.28\sqrt{0.44\left(1-0.44\right)+0.50\left(1-0.50\right)}\right]}^2}{{\left(0.44-0.50\right)}^2} $$

*n* = 2055 households for each

Therefore the sample size was 2050 for each (4110 total). This sample was compared with the sample that was determined for food insecurity objectives that was calculated using StatCal of Epi Info utility with *P* = 50 % (the possible maximum sample size) and a precision level of 0.02. The sample was found to be 2396 (1198 for each category) at 95 % confidence level. Multistage sampling technique was implemented to reach and select the final study units. In the first place the two zones (east and west Gojjam) were selected purposely by taking into account the availability and absence of the productive safety net program in the two zones. This is because areas covered by the productive safety net program are considered as food insecure (the three districts in east Gojjam zone in this case) and west Gojjam zone is considered as a food surplus area (based on highly productive nature of the zone) by the regional government. Six districts from the two zones (three from each zone) were selected. The three districts from east Gojjam zone (Enebsie Sar Midir, Goncha Siso Enesie, and Shebel Berenta) covered by the safety net program were purposely selected. Three equal numbers of districts (Mecha, North Achefer, and Jabi Tehinan) in the west Gojjam zone were selected randomly from the total 14 districts. The two zones are more comparable in many socio-cultural characteristics than the other zones of the region.

Once the districts were identified, kebeles (the smallest administrative unit in the country) from those districts with the program were selected randomly and included in the study. The kebeles were selected based on agro-ecological zones and urban rural settings. Four town kebeles, three rural high land kebeles, eleven rural mid-land kebeles, and six rural lowland kebeles were selected randomly.

Then, the total sample size was divided proportionally to the kebele households. The households from these kebeles were selected using a systematic random sampling technique using household registration as a sampling frame. For the case of east Gojjam zone, safety net program registration was used as a sampling frame. The total number of households in each kebele was divided by the allocated sample size to get the sampling interval. When there was more than one mother in the same household, one mother was selected by lottery method.

### Data collection tools and techniques

Structured questionnaires, adopted from different standard questionnaires [27, 28] and developed by the authors, were used to collect the data. Some of the variables adopted from the EDHS questionnaire include age of the mother, marital status, educational level, family size, occupation, household monthly income and housing conditions. Variables like household (HH) head, female authority and agro-ecological zone were prepared and included in the questionnaire by the authors. Household food security (access) information was collected by using the questionnaire adopted from the Household Food Insecurity Access Scale (HFIAS) measurement tool which is developed by Food and Nutrition Technical Assistant Project (FANTA) [29]. The questionnaire was translated in to the local language (Amharic). Beside the translation of the questionnaire from English to Amharic and back to English, a pre-test was done on 120 subjects to check if they understood it easily or not. After the pre-test was done, a detailed demonstration was given to data collectors especially on ways how to explain the questionnaire to the respondent.

### Operational definitions

Food secure - household experiences none of the food insecurity conditions, or just experiences worry, but rarely [29].

Mildly food insecure - household worries about not having enough food sometimes or often, and/or is unable to eat preferred foods, and/or eats a more monotonous diet than desired and/or some foods considered undesirable, but only rarely [29].

Moderately food insecure - household sacrifices quality more frequently, by eating a monotonous diet or undesirable foods sometimes or often, and/or has started to cut back on quantity by reducing the size of meals or number of meals, rarely or sometimes [29].

Severely food insecure - household has graduated to cutting back on meal size or number of meals often, and/or experiences any of the three most severe conditions (running out of food, going to bed hungry, or going a whole day and night without eating). In other words, any household that experiences one of these three conditions even once in the last four weeks [29].

### Data quality control

To assure the quality of the data and to make sure that all assessment team members were able to administer the questionnaires properly, a total of five days rigorous training of enumerators and supervisors was given. Before the actual data collection work, data collectors and supervisors carried out role play practices and then had field pre-test activities. The data collectors and supervisors were university graduate BSc holders. At the end of every data collection day, each questionnaire was examined for completeness and consistency by the supervisors and the principal investigator, and pertinent feedback was given to the data collectors and supervisors.

### Data management and analysis

The data were coded, entered and cleaned by Epi-Info 2000 version 3.5.3 and transported to SPSS version 20. Descriptive summaries such as frequencies, proportions, percentages, mean, standard deviations and prevalence were determined. Excel was used to determine food insecurity prevalence and to identify the four categories (food secure, mildly food insecure, moderately food insecure and severely food insecure) by using IF OR/AND logical test function formula. For determinant variable identification, first bivariate logistic regression analyses were carried out to identify candidate variables for multivariate model at *P*-value < 0.25. Then, to identify the predictors of food insecurity variables that were significantly associated with food insecurity in the bivariate models were entered in the multivariate logistic regression model. At this step, model fitness and the presence of multicollinearity were assessed. The covariate also categorized into socio-demographic and environmental determinants. The model fitness was checked by observing the difference of the -2 log likelihood ratio between the model with only the constant and with the predictors. The significance of each predictor in the equation was also assessed by Wald statistics test at a significance level of *P*-value < 0.05. Few variables were excluded from the last model due to instability of the model with their presence and their high correlation (maternal occupation; hose wife versus farmer, *r* = 0.934).

## Results

### Socio-demographic characteristics

Among the 4110 households visited, 3964 respondents (with a response rate of 96.45 %) gave complete responses. The age distribution of the respondents range from 15–49 years and the majority were in 20–29 year age group (53.6 %) and the least were in 15–19 year age group (1.3 %). One thousand nine hundred eighty five (50.10 %) respondents were from East Gojjam and the rest 1979 (49.90 %) were from west Gojjam. The majority (85.50 %) of the head of households were men. About 86 % of the respondents were rural residents (Table 1). The agro-ecological zone distribution of the respondents was 363 (9.20 %) from the highlands (*Dega*) elevation of > 2400 m, 2373 (59.90 %) from medium highland (*Woyna Dega*) elevation of 1500–2400 m, and 1228 (31 %) from lowland (*Kola*) elevation of < 1500 m.

**Table 1:** Socio-demographic characteristics of the study population in East Gojjam (*n* = 1985) and West Gojjam (*n* = 1979) zones of Amhara region, 2013

Variables	East Gojjam		West Gojjam		Total	
	Number	Percent	Number	Percent	Number	Percent
Household head:						
Female	291	14.7	277	14.0	568	14.3
Male	1694	85.3	1702	86.0	3396	85.7
Marital status:						
Married	1689	85.1	1721	87.0	3410	86.0
Divorced	231	11.6	157	7.9	388	9.8
Widowed	46	2.3	57	2.9	103	2.6
Separate	7	0.4	20	1.0	27	0.7
Single	12	0.6	24	1.2	36	0.9
Family size:						
2-4	979	49.3	893	45.1	1872	47.2
≥5	1006	50.7	1086	54.9	2092	52.8
Maternal educational level:						
No formal education	1722	86.8	1559	78.8	3281	82.8
Have a formal education	263	13.2	420	21.2	683	17.2
Paternal educational level:						
No formal education	1322	76.6	1205	67.5	2527	72.0
Have a formal education	404	23.4	580	32.5	984	28.0
Maternal occupation:						
Housewife	765	38.5	1042	52.7	1807	45.6
Farmer	1021	51.8	570	28.8	1591	40.1
Private organization employee	4	0.2	17	0.9	21	0.5
Merchant	188	9.5	245	12.4	433	10.9
Government employ	12	0.6	63	3.2	75	1.9
Day labourer	21	1.1	78	3.9	99	2.5
Others	37	1.9	37	1.9	74	1.9
Paternal occupation:						
Farmer	1514	76.3	1244	63.0	2758	69.6
Merchant	178	9.0	305	15.4	483	12.2
Private organization employee	9	0.5	55	2.8	64	1.6
Day labourer	28	1.4	90	4.6	118	3.0
Government employee	32	1.6	111	5.6	143	3.6
Others	11	0.6	52	2.6	63	1.6
Residential place:						
Rural	1917	96.9	1499	75.7	3416	86.2
Urban	68	3.4	480	24.3	548	13.8
Family monthly income:						
1^st^ quartile (80–400 ETB)	797	40.2	429	21.7	1226	30.9
2^nd^ quartile (403–560 ETB)	468	23.6	294	14.9	762	19.2
3^rd^ quartile (561–800 ETB)	486	24.5	656	33.1	1142	28.8
4^th^ quartile (810–9000 ETB)	234	11.8	600	30.3	834	21.0

### Level of food insecurity (access)

**Fig. 1:**
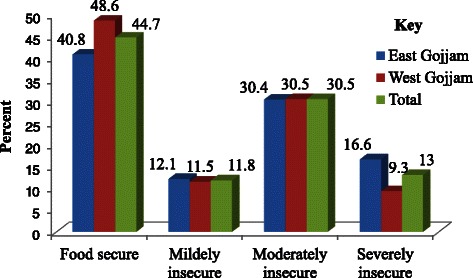
Comparison of food insecurity status between the two zones, East and West Gojjam Zones of Amhara Region 2013

Generally, the prevalence of food insecurity (access) was 55.30 % (95 % CI: 53.80, 56.80) and of these, 13 % (95 % CI: 11.90, 14.00) were severely food insecure. Comparing food insecurity levels within the two zone population showed that nearly sixty percent (59.20 %) (95 % CI: 57 %, 61.4 %) of the east Gojjam and 51.30 % (95 % CI: 49.1 %, 53.5 %) of west Gojjam households were food insecure (Fig. 1). Of the total respondents 1692 (42.70 %) households experienced anxiety and uncertainty about food supply. Two thousand one hundred eighty three (55.07 %) respondents encountered insufficient quality that includes variety and preferences of the type of food, whereas 1720 (43.39 %) of the population experienced insufficient food intake and its physical consequence.

### Determinants of food insecurity

Many variables assumed to be determinants of food insecurity status of the households were tested for their significance prediction on the outcome variable. The logistic regression analysis on the socio-demographic characteristics showed that maternal education status was the most significant predictor of food insecurity in the west Gojjam zone population but not for the east Gojjam zone. On the other hand, household family size was found to be a significant predictor in the east Gojjam zone population but not for west Gojjam. Maternal occupation types, paternal occupation types and family monthly income were the other statistically significant predictors for food insecurity in both study communities. In the same way from environmental characteristics, residential area, agro-ecological zones, house roof made of corrugated iron sheet and number of rooms were significant predictors of food insecurity in both communities. Availability of livestock was also found to be a statistically significant predictor of food insecurity in the two communities.

The odds of illiterate mothers in west Gojjam zone being food insecure were 1.388 times higher than mothers who had formal education exposure (AOR = 1.388, 95 % CI: 1.011, 1.905, with *P* < 0.01). The odds of households who have a family size of < 5 were 0.641 times lower than households who have family size ≥ 5 (AOR = 0.641, 95 % CI: 0.513, 0.801, with *P* < 0.001) in east Gojjam zone; however, family size was not found to be significant predictor in west Gojjam zone. Households in which women did not engage in farming activities were more likely to be food insecure compared to their counter parts in east Gojjam zone (AOR = 4.795, 95 % CI: 3.753, 6.125, *P* < 0.001) but not for west Gojjam. The quartiles of family monthly income; first (80–400 Ethiopian Birr (ETB)), second (403–560 EB) and third (561–800 ETB) quartiles compared with fourth quartile (810–9000 ETB) were statistically significant predictors in both study communities (Table 2 & 3). The odds of rural residents were 3.201 times higher than urban residents to be food insecure (AOR = 3.201, 95 % CI: 1.832, 5.594, *P* < 0.001) in east Gojjam zone and 2.425 times higher in west Gojjam zone (AOR = 2.425, 95 % CI: 1.797, 3.272, *P* < 0.001) (Table 4 & 5). There was no difference for food insecurity between middle highland and lowland dwellers. However, households living in the highlands were 2.193 times more likely to become food insecure compared to low lands (AOR = 2.193, 95 % CI: 1.348, 3.569, *P* < 0.001) in east Gojjam zone (Table 4). On the other hand, in west Gojjam zone both highland and middle highland populations were more likely to become food insecure compared to lowland populations (AOR = 3.669 & 3.094, 95 % CI: 2.442, 5.513 & 2.210, 4.331, *P* < 0.001) respectively (Table 5). Those households who did not have livestock were 1.553 times more likely to become food insecure in east Gojjam zone and 1.568 times in west Gojjam zone compare with households who have livestock (AOR = 1.553 & 1.568, 95 % CI: 1.160, 2.078 & 1.183, 2.080, *P* < 0.01) respectively (Table 4 & 5).

**Table 2:** Binary logistic regression analysis of socio-demographic variables predicting the odds of food insecurity among households (*n* = 1985) in East Gojjam Zones of Amhara region, 2013

Characteristics	Food security status		Crude OR (95 % CI)	Adjusted OR (95 % CI)
	Insecure *N* (%)	Secure *N* (%)		
Household head:				
Female ^R^	162 (55.7)	129 (44.3)	1	1
Male	1013 (59.8)	681 (40.2)	1.185 (0.922, 1.522)	1.215 (0.565, 2.612)
Female authority:				
Yes	1146 (59.6)	778 (40.4)	1.625 (0.975, 2.709)	1.320 (0.747, 2.381)
No ^R^	29 (47.5)	32 (52.5)	1	1
Maternal education:				
No formal education	1041 (60.5)	681 (39.5)	1.472 (1.134, 1.9.9)^**^	1.058 (0.746, 1.499)
Formal education	134 (51.0)	129 (490)	1	1
Paternal education:				
No formal education	812 (61.4)	510 (38.6)	1.386 (1.107, 1.735)^**^	1.083 (0.828, 1.418)
Formal education	212 (53.5)	188 (46.5)	1	1
Family size:				
2–4	541 (55.9)	432 (44.1)	0.762 (0.637, 0.912)^***^	0.641 (0.513,0.801)^***^
≥ 5	628 (62.4)	378 (37.6)	1	1
Maternal occupation				
Farmer:	491 (48.1)	530 (51.9)	1	1
Yes				
No	684 (71.0)	280 (29.0)	2.637 (2.190, 3.174)^***^	4.795 (3.753, 6.125)^***^
Merchant:				
Yes	89 (47.3)	99 (52.7)	1	1
No	1086 (60.4)	711 (39.6)	1.699 (1.257, 2.297)^***^	1.638 (1.015, 2.643)^*^
Day labourer:				
Yes	12 (57.1)	9 (42.9)	1	1
No	1163 (59.2)	801 (40.8)	1.089 (0.457,2.596)	0.928 (0.186, 4.626)
Paternal occupation				
Farmer:				
Yes	913 (60.3)	601 (39.7)	1	1
No	262 (55.6)	209 (44.4)	0.825 (0.670, 1.017)	0.528 (0.321, 0.869)^*^
Merchant:				
Yes	96 (53.9)	82 (46.1)	1	
No	1079 (59.7)	728 (40.3)	1.265 (0.929, 1725)	0.770 (0.459, 1.292)
Day labourer:				
Yes	17 (60.7)	11 (39.3)	1	1
No	11.58 (59.2)	799 (40.8)	0.938 (0.437, 2.013)	0.720 (0.286, 1.813)
Monthly income:				
1^st^ quartile	501 (62.9)	296 (37.1)	1.908 (1.422, 2.561)^**^	2.756 (1.902, 3.993)^***^
2^nd^ quartile	298 (63.7)	170 (36.3)	1.976 (1.437, 2.717)^**^	1.897 (1.299, 2.771)^**^
3^rd^ quartile	266 (54.7)	220 (45.3)	1.363 (0.997, 1.863)	1.070 (0.743, 1.543)
4^th^ quartile ^R^	110 (47.0)	12 (53.0)	1	1

^*^ = 0.05, ^**^ = 0.01, ^***^ = 0.001 Significant level, R = Reference group

**Table 3:** Binary logistic regression analysis of socio-demographic variables predicting the odds of food insecurity among households (*n* = 1979) in West Gojjam Zones of Amhara region, 2013

Characteristics	Food security status		Crude OR (95 % CI)	Adjusted OR (95 % CI)
	Insecure *N* (%)	Insecure *N* (%)		
Household head:				
Female ^R^	184 (66.4)	93 (33.6)	1	1
Male	833 (48.9)	869 (51.1)	0.484 (0.371, 0.633)^***^	1.041 (0.646, 1.680)
Female authority:				
Yes	975 (51.6)	915 (48.4)	192 (0.779, 1.825)	1.032 (0.657, 1.623)
No ^R^	42 (47.2)	47 (52.8)	1	1
Maternal education:				
No formal education	848 (54.4)	711 (45.6)	1.771 (1.423, 2.205)^***^	1.388 (1.011, 1.905)^*^
Formal education	169 (40.2)	251 (59.8)	1	1
Paternal education:				
No formal education	637 (52.9)	568 (47.1)	1.589 (1.300, 1.941)	1.110 (0.864, 1.457)
Formal education	240 (41.4)	340 (58.6)	1	1
Family size:				
2–4	485 (84.3)	408 (45.7)	1.238 (1.037, 1.478)^*^	1.088 (0.878, 1.3490
≥ 5	532 (49.0)	554 (51.0)	1	1
Maternal occupation				
Farmer:				
Yes	271 (47.5)	299 (52.5)	1	1
No	746 (52.9)	663 (47.1)	1.241 (1.022, 509)^*^	1.237 (0.980, 1.560)
Merchant:				
Yes				
	138 (56.3)	107 (43.7)	1	1
No	879 (50.7)	855 (49.3)	0.797 (0.609, 1.044)	0.375 (0.251, 0.559)^***^
Day labourer:				
Yes	63 (80.8)	15 (19.2)	1	1
No	954 (50.2)	947 (49.8)	0.240 (0.136, 0.424)^***^	0.460 (0.191, 1.106)
Paternal occupation				
Farmer:				
Yes	642 (51.6)	602 (48.4)	1	1
No	374 (51.2)	357 (48.8)	0.982 (0.818, 1.179)	0.954 (0.683, 1.333)
Private employee:				
Yes	22 (40.0)	32 (60.0)	1	
No	994 (51.8)	925 (48.2)	1.612 (0.933, 2.785)	
Merchant:				
Yes	115 (37.7)	190 (62.3)	1	1
No	901 (54.0)	769 (46.0)	1.936 (1.506, 2.487)^***^	1.866 (1.285, 2.712)^**^
Day labourer:				
Yes	68 (75.6)	22 (24.4)	1	1
No	948 (50.3)	937 (49.7)	0.327 (0.201, 0.534)^***^	0.436 (0.246,0.772)^**^
Monthly income:				
1^st^ quartile	288 (67.1)	141 (32.9)	4.442 (3.408, 5.790)^***^	3.232 (2.366, 4.418)^***^
2^nd^ quartile	183 (62.2)	111 (37.8)	3.585 (2.677, 4.801)^***^	2.618 (1.892, 3.622)^***^
3^rd^ quartile	357 (54.4)	299 (45.6)	2.596 (2.061, 3.271)^***^	2.177 (1.691, 2.803)^***^
4^th^ quartile ^R^	189 (31.5)	411 (68.5)	1	1

^*^ = 0.05, ^**^ = 0.01, ^***^ = 0.001 Significant level, R = Reference group

**Table 4:** Binary logistic regression analysis of environmental variables predicting the odds of food insecurity among households (*n* = 1985) in East Gojjam zone of Amhara region, 2013

Characteristics	Food security status			
	Insecure *N* (%)	Secure *N* (%)	Crude OR (95 % CI)	Adjusted OR (95 % CI)
Residential area:				
Urban ^R^	22 (32.4)	46 (67.6)	1	1
Rural	1153 (60.1)	764 (39.9)	3.156 (1.883, 5.288)***	3.201 (1.832, 5.594)***
Agroecology:				
Kolla ^R^	657 (63.8)	372 (36.2)	1	1
Dega	67 (72.0)	26 (28.0)	1.459 (0.912, 2.335)	2.193 (1.348, 3.509)**
Woynadega	451 (52.3)	412 (47.7)	0.620 (5.515, 0.745)***	0.843 (0.679, 1.066)
Latrine availability:				
Yes ^R^	753 (58.7)	530 (41.3)	1	1
No	422 (60.1)	280 (39.9)	1.061 (0.879, 1.280)	0.934 (0.765, 1.141)
Roof made of:				
CIS ^R^	926 (55.6)	739 (44.4)	1	1
Grass roofed	249 (77.8)	71 (22.2)	2.799 (2.113, 3.706)**	1.822 (1.288, 2.705)**
Number of rooms:				
1	399 (68.8)	181 (31.2)	4.509 (2.630, 7.732)***	2.237 (1.250, 4.004)**
2	463 (56.3)	359 (43.7)	2.638 (1.556, 4.444)***	2.165 (1.259, 3.725)**
3	291 (56.4)	225 (43.6)	2.645 (1.544, 4.534)***	2.279 (1.315, 3.948)**
4 ^R^	22 (32.8)	45 (67.2)	1	1
Kitchen availability:				
Yes	642 (51.5)	605 (48.5)	1	1
No	533 (72.2)	205 (27.8)	2.450 (2.015, 2.980)***	1.951 (1.567, 2.429)***
Have livestock:				
Yes ^R^	985 (58.7)	694 (41.3)	1	1
No	190 (62.1)	116 (37.9)	1.154 (0.898, 1.483)	1.553 (1.160, 2.078)**

** = 0.01, *** = 0.001 Significant level, R = Reference group

**Table 5:** Binary logistic regression analysis of environmental variables predicting the odds of food insecurity among households (*n* = 1979) in West Gojjam zone of Amhara region, 2013

Characteristics	Food security status			
	Insecure *N* (%)	Secure *N* (%)	Crude OR (95 % CI)	Adjusted OR (95 % CI)
Residential area:				
Urban ^R^	199 (41.5)	281 (58.5)	1	1
Rural	818 (54.6)	68 (45.5)	1.696 (1.377, 2.089)**	2.425 (1.797, 3.272)**
Agroecology:				
Kolla ^R^	59 (29.6)	140 (70.4)	1	1
Dega	174 (64.4)	96 (35.6)	4.301 (2.902, 6.373)**	3.669 (2.442, 5.513)**
Woynadega	784 (51.9)	726 (48.1)	2.562 (1.860, 3.531)**	3.094 (2.210, 4.331)**
Latrine availability:				
Yes ^R^	833 (49.6)	848 (50.4)	1	1
No	184 (61.7)	114 (38.3)	11.643 (1.277, 2.115)**	1.432 (1.093, 1.875)*
Roof made of:				
CIS ^R^	998 (51.0)	958 (49.0)	1	1
Grass roofed	19 (82.6)	4 (17.4)	4.560 (1.546, 13.452)**	2.584 (0.856, 7.794)
Number of rooms:				
1	362 (61.9)	223 (38.1)	3.996 (2.65, 6.008)**	2.956 (1.922, 4.548)**
2	336 (46.0)	395 (54.0)	2.094 (1.404, 3.122)**	1.769 (1.168, 2.678)*
3	280 (53.0)	248 (47.0)	2.779 (1.845, 4.186)**	2.553 (1.673, 3.894)**
4 ^R^	39 (28.9)	96 (71.1)	1	1
Kitchen availability:				
Yes	651 (48.5)	692 (51.5)	1	1
No	366 (57.5)	270 (42.5)	1.441 (1.191, 1.743)**	1.149 (0.936, 1.410)
Have livestock:				
Yes ^R^	729 (51.8)	679 (48.2)	1	1
No	288 (50.4)	283 (49.6)	0.948 (0.780,1.151)	1.568 (1.183, 2.080)*

* = 0.01, ** = 0.001 Significant level, R = Reference group

Some variables were significantly associated with food insecurity levels of the households in the first model: however the association was not persistent in the second model when other variables were added. For example, there was a significant association between household head and food insecurity in the bivariate analysis in east Gojjam zone although it disappeared in the multivariate model. However, there was a substantial difference between female headed and male headed households. It was found that, from the total female headed households, 59.3 % were in the first quartile and from the total male headed households 26.2 % were in the first quartile. On the other hand, from the total female headed households only 8.6 % were in the 4^th^ quartile where as 23.1 % of the class was male headed households.

## Discussion

The present study determined the level of food insecurity and identified the associated factors at the household level. Almost sixty percent (59.20 %) of the households in east Gojjam zone were food insecure. Although the same tool to categorize food security and insecurity was not used at the beginning of the project, at this level there was a 40 % improvement from the food insecure households. According to the present study, more than half of the households from West Gojjam zone (which is categorized as a food surplus area) were food insecure. This indicates that both zones are at higher risk of food insecurity and any intervention related to a food security program should address both study areas. Forty two percent of the food insecure households experienced anxiety and uncertainty about the household food and 55.07 % experienced insufficient quality which may include limited choice and preferences. This shows that one or more household members ate food that was socially or personally undesirable and of limited diversity. About 43.39 % of the food insecure household experience insufficient food intake or have no food of any kind to eat.

Taking in to account the total prevalence of food insecurity, the findings of this study were similar to endogenous study results reported from Addis Ababa (58.16 %) and Jimma (54.8 %; the transient food insecurity levels of adolescents) [8, 17]. They are also the same as the findings reported from a study done in Sidama, Southern Ethiopia (54.1 %) [18]. However, the prevalence was lower than study finding from Farta woreda, Northwest Ethiopia (70.7 %) [16]. This shows that there is a variation in the magnitude of the level of food insecurity in different localities within the country. To get a good picture of the level of food insecurity at a national level, doing similar studies on a larger scale is important. Another possible explanation for the difference could be seasonal variation because seasonal variation has paramount significance on the food security of certain communities.

In addition to the prevalence of food insecurity, the study identified different determinants for the presence of food insecurity in the study communities. The level of formal education of women was found to be a significant predictor of household food insecurity in west Gojjam zone; however this was not true for east Gojjam. Those who did not attain formal education were more likely to be food insecure compared to those who did. This result is supported by studies done in Canada, Nigeria and Ethiopia [10, 16, 17, 30]. This could be explained by the probability that more educated mothers will ensure family members and will likely be more familiar with modern technology and other developments [17]. Educated women have a better chance of managing their farm by adopting improved practices which in turn increase total yield. It is assumed that educated households often tend to adopt new skills and ideas which in turn have positive effects on food security.

It was also found that households where women did not engage in different jobs compared to those who did, had different probabilities of being food insecure. As supported by other studies done in Addis Ababa and Ghana, those households where women engaged in market and agricultural activities were less likely to be food insecure compared to those who were unemployed [17, 31]. Households could diversify their income by utilizing indifferent job opportunities. Off-farm revenue generating activities have a paramount significance in the diversification of sources of farm incomes [31]. It is expected that when household members are involved in different job opportunities and diversified their income sources, they can improve their wealth status and enable the household to become food secure.

The other significant predictor of household food insecurity was household monthly income. It was found that households with the lowest quartile of monthly income were more likely to be food insecure compared to the fourth quartile. This was true for both study populations. The lower the income, the higher is the risk of exposure to food insecurity. This finding is consistent with study results reported from Ontario (Canada), South Africa, and Nigeria [10, 30, 32, 33]. Similar to the study in Farta woreda (Ethiopia), in the present study households who had no livestock animals were more likely to become food insecure compared to those households who have livestock animals [16] and this was the other variable significantly associated with food insecurity in both study communities. This might be due to the fact that rural households accumulate their wealth in terms of livestock and purchase food crops by selling these livestock when they faced food shortages.

According to the study, residential area was other determinant of household food insecurity. Rural households were more likely to be food insecure than urban, however, the rural residents have a better chance to access agricultural products. In urban areas, household food security depends on household income, work opportunities and an efficient food market system [22]. It might be due to these facts that urban households were less likely to be food insecure compared to rural, although the latter have a direct exposure to agricultural products.

Vulnerability to food insecurity is a common phenomenon along the semi-arid lowlands and degraded high lands of Ethiopia where rural households rely on rain-fed agriculture [34]. In the present study, households in the highland agro-ecological zone were more likely to be food insecure than lowland zones but there was no difference between lowland and middle highland households. The findings are similar to studies done in different parts of Ethiopia in which households in the highland agro-ecological zone are more likely food insecure compared to households in lowlands ([34], Negatu Workneh: Reasons for food insecurity of farm households in south Wollo, Ethiopia: Explanation at grassroot, unpublished). Most highland areas are mountainous and prone to soil erosion and degradation, and these could be risk factors for the reduction of agricultural productivity. In Ethiopia, soil erosion and degradation are common events since there are no sustainable soil and water management practices. Soil erosion and degradation substantially affect food security [35]. On the other hand in lowlands, households prefer to cultivate cash crops and to purchase food from markets [34] thus, they are less likely to be food insecure.

### Limitation of the study

Use of the cross-sectional method limits the study to showing seasonal variability of food insecurity in the study areas. There was no base line data compiled using a similar tool when project area was initially categorized as food insecure. Therefore, it is difficult to conclude whether the improvement is significant or not.

## Conclusion

There is a high prevalence of food insecurity in both study areas and there are also different determinant factors. Therefore, the food security intervention programme in the country should reconsider and give emphasis to household education, as it largely contributes to working efficiency, competency, diversification of income, and adoption of new technology. This means that educated household heads play a significant role in shaping household income. Attaining food security in the highlands of Ethiopia requires adoption of a mixed strategy that includes engaging substantially in non-staple cash enterprises like livestock rearing, cash crops, and trade implying diversification based on local resources and market opportunities. The already started soil and water conservation activities should be strengthened and continued to prevent soil erosion and degradation. As monthly household income and food insecurity are significantly associated, research into improved technical skills and the education of farmers to make them competitive in the current farming system and thus in generating income should be a government priority.

## Abbreviations

EDHS, Ethiopian Health and Demographic Survey; ETB, Ethiopian Birr; FANTA, Food and Nutrition Technical Assistant project; FAO, Food and Agriculture Organization; HFIAS, household food insecurity access scale; HH, household; IRB, Institutional Review Board; Kcal/cap/day, kilocalorie per capita per day

## References

[1] Seligman H, Laraia B, Kushel M (2010). Food Insecurity is Associated with Chronic Disease among Low-income NHANES Participants. J Nutr.

[2] Willows N, Veugelers P, Raine K, Kuhle S (2011). Associations between Household Food Insecurity and Health Outcomes in the Aboriginal Population (excluding reserves).

[3] Keino S, Plasqui G, van den Borne B (2014). Household Food Insecurity Access: A Predictor of Overweight and Underweight among Kenyan Women. Agric Food Secur.

[4] World Food Summit (1996). Rome Declaration on World Food Security and World Food Summit Plan of Action, Rome.

[5] Endalew B, Muche M, Tadesse S (2015). Assessment of food security situation in Ethiopia: A Review. Asian J Agric Res.

[6] Kuwornu J, Suleyman D, Amegashie D (2014). Comparative Analysis of Food Security Status of Farming Households in the Coastal and the Forest Communities of Central Region of Ghana. Asian J Empir Res.

[7] Napoli M, De Muro P, Matteo M: Master in Human Development and Food Security: Towards a Food Insecurity Multidimensional Index (FIMI). 2010/2011.

[8] Belachew T, Lindstrom D, Gebremariam A, Jira C, Hattori MK, Lachat C, Huybregts L, Kolsteren P (2012). Predictors of Chronic Food Insecurity among Adolescents in Southwest Ethiopia: A Longitudinal Study. BMC Public Health.

[9] Carter NK, Lanumata T, Kruse K, Gorton D: What Are the Determinants of Food Insecurity in New Zealand and Does This Differ for Males and Females? Aust N Z J Public Health. 2010, 34(5). doi: 10.1111/j.1753-6405.2010.00615.x. 10.1111/j.1753-6405.2010.00615.x 21134063

[10] Willows N, Veugelers P, Raine K, Kuhle S (2008). Prevalence and Sociodemographic Risk Factors Related to Household Food Security in Aboriginal peoples in Canada. Public Health Nutr.

[11] Furness B, Simon P, Wold C, Asarian-Anderson J (2004). Prevalence and Predictors of Food Insecurity among Low-income Households in Los Angeles County. Public Health Nutr.

[12] Mitiku A, Fufa B, Tadese B (2012). Emperical analysis of the determinants of rural households food security in Southern Ethiopia: The case of Shashemene District. Basic Res J Agric Sci Rev.

[13] FAO (2015). Regional overview of food insecurity: African food insecurity prospects brighter than ever. Accra, FAO.

[14] FAO, IFAD, WFP (2014). The State of Food Insecurity in the World 2014. Strengthening the enabling environment for food security and nutrition. Rome, FAO.

[15] Nagothu U, Tesfai M, Adugna A (2015). Food Security and Development, Country case studies: Status and trends of food security in Ethiopia.

[16] Endale W, Mengesha ZB, Atinafu A, Adane AA (2014). Food Insecurity in Farta District, Northwest Ethiopia: A Community Based Cross-sectional Study. BMC Res Notes.

[17] Gezimu G (2012). Determinants of Food Insecurity among Households in Addis Ababa City, Ethiopia. Interdiscip Descr Complex Sys.

[18] Regass N (2011). A Small Holder Farmers Coping Strategies to Household Food Insecurity and Hunger in Sidama Woreda Southern Ethiopia. EJESM.

[19] Gucciardi E, Vogt J, DeMelo M, Stewart D (2009). Exploration of the Relationship Between Household Food Insecurity and Diabetes in Canada. Diabetes Care.

[20] Carter A, Dubois L, Tremblay S, Taljaard M (2012). Local Social Environmental Factors are Associated with Household Food Insecurity in a Longitudinal Study of Children. BMC Public Health.

[21] Vozoris TN, Tarasuk SV (2003). Household Food Insufficiency Is Associated with Poorer Health. J Nutr.

[22] Adenew B (2004). The Food Security Role of Agriculture in Ethiopia. Electron J Agric Dev Econ.

[23] Devereux S, Sabate-Wheeler R, Tefera M, Taye H (2006). Ethiopia’s Productive Safety Net Programme (Psnp): Trends in PSNP Transfers Within Targeted Households, Final Report.

[24] Amhara Bureau of Finance and Economic Development (2014). Population Size by Sex and Age Group and Urban and Rural.

[25] Amhara Regional State (2002). A Strategic Plan for the Sustainable Development, Conservation, and Management of the Woody Biomass Resources, Final Report.

[26] Ayalew D, Tesfaye K, Mamo G, Yitaferu B, Bayu W (2012). Variability of rainfall and its current trend in Amhara region, Ethiopia. Af J Agric Res.

[27] Central Statistical Agency [Ethiopia] and ICF International (2012). Ethiopia Demographic and Health Survey 2011. Addis Ababa, Ethiopia and Calverton, Maryland, USA: Central Statistical Agency and ICF International.

[28] World Food Program: Emergency Food Security Assessment Handbook, Second ed. 2009.

[29] Coates J, Swindale A, Bilinsky P (2007). Household Food Insecurity Access Scale (HFIAS) for Measurement of Household Food Access: Indicator Guide (v. 3).

[30] Babutunde O, Omotesho A, Sholotan S (2007). Factors Influencing Food Security Status of Rural Farming Households in North Central Nigeria. Agric J.

[31] Aidoo R, Mensah J, Tuffour T (2013). Determinants of Household Food Security in The Sekyere-Afram Plains District of Ghana.

[32] Tarasuk V, Vogt J (2009). Household Food Insecurity in Ontario. Can J Public Health.

[33] Sekhampu T (2013). Determinants of the Food Security Status of Households Receiving Government Grants in Kwakwatsi, South Africa. Mediterranean J Soc Sci.

[34] Yirgu B (2013). An Agro-Ecological Assessment of Household Food Insecurity in Mid-Deme Catchment, South-western Ethiopia. Global Adv Res J Geogr Reg Plann.

[35] Beyene F, Muche M (2010). Determinants of Food Security among Rural Households of Central Ethiopia: An Empirical Analysis. Q J Int Agric.

